# Experimental infection of chickens, Pekin ducks, Eurasian wigeons and Barnacle geese with two recent highly pathogenic avian influenza H5N1 clade 2.3.4.4b viruses

**DOI:** 10.1080/22221751.2024.2399970

**Published:** 2024-09-02

**Authors:** Luca Bordes, Evelien A. Germeraad, Marit Roose, Nadiah M. H. A. van Eijk, Marc Engelsma, Wim H. M. van der Poel, Sandra Vreman, Nancy Beerens

**Affiliations:** a Wageningen Bioveterinary Research (Wageningen University and Research), Lelystad, The Netherlands; b Department Biomolecular Health Sciences, Division of Pathology, Faculty of Veterinary Medicine, Utrecht University, Utrecht, The Netherlands

**Keywords:** Avian influenza clade 2.3.4.4b, chicken, Pekin duck, Eurasian wigeon, Barnacle geese

## Abstract

Multiple genotypes of highly pathogenic avian influenza (HPAI) H5 clade 2.3.4.4b viruses have caused epizootics in wild birds and poultry. The HPAI H5N1 genotype C virus caused a modest epizootic, whereas the occurrence of the HPAI H5N1 genotype AB virus in 2021 resulted in the largest avian influenza epizootic in Europe to date. Here we studied the pathogenicity of two HPAI H5N1 viruses by experimentally infecting chickens, Pekin ducks, Eurasian wigeons and Barnacle geese. Our study demonstrates that pathogenicity of the H5N1-2021-AB virus is lower in Pekin ducks, Eurasian wigeons and Barnacle geese compared to the H5N1-2020-C virus, whereas virus shedding was high for both viruses. After inoculation with H5N1-2021-C viral antigen expression was higher in the brain of Pekin ducks, Eurasian wigeons and Barnacle geese, which caused higher mortality compared to inoculation with H5N1-2021-AB virus. Subclinical infections occurred in Pekin ducks and Eurasian wigeons and mortality was reduced in Barnacle geese after inoculation with H5N1-2021-AB virus while H5N1-2020-C virus caused high morbidity and mortality in these species. This H5N1-2021-AB virus trait may have contributed to efficient spread of the virus in wild bird populations. Therefore, high mortality, virus shedding and long-lasting viral antigen expression found in Barnacle geese may have increased the risk for introduction into poultry.

## Introduction

Highly pathogenic avian influenza (HPAI) H5-viruses have a long history in Asia and Europe, and are distributed by migratory wild birds [1,2]. The first HPAI virus introduction from poultry to wild birds was reported in 1996 in southern China with the A/Goose/Guangdong/1/1996 H5N1 strain marking the genesis of the Eurasian H5 Goose/Guangdong (Gs/Gd) lineage. Shortly after this initial introduction, the first HPAI spillback caused an outbreak in Hong Kong (1997), resulting in substantial mortality in poultry and eighteen confirmed human cases [3]. Prior to the emergence of the Gs/Gd lineage, HPAI viruses were largely confined to poultry, but the Gs/Gd lineage showed an exceptionally broad host range involving commercial poultry, wild birds, mammals and humans [4].

The evolutionary trajectory of the Gs/Gd lineage, characterized by reassortments (with unknown influenza viruses) and mutations, led to its diversification into numerous genotypes [5]. Since 2003, HPAI of the Gs/Gd lineage is enzootic in Asia causing repeated outbreaks in poultry [6]. Notably, in 2005, a HPAI H5N1 clade 2.2 virus caused significant wild bird mortality, particularly among bar headed geese in Qinghai Lake, China [7]. Subsequently, this Gs/Gd H5N1 clade 2.2 virus was detected in Europe, initially in backyard farms and wild birds in Turkey, Romania and Croatia, continuing to spread to 24 European countries until 2009 [8]. Wild bird mortality in Europe primarily affected Mute Swans (*Cygnus olor*) and Whooper Swans (*Cygnus cygnus*) [9].

Various introduction routes were considered for the European incursion of the HPAI H5N1 virus in 2005, including the involvement of long-distance migratory birds [10–12]. Migratory birds of the order Anseriformes, which includes species of ducks, geese and swans likely played an important role in virus dispersion. In particular, infected birds that do not display severe disease signs, such as Eurasian wigeons (*Anas Penelope)* may have been involved in virus spread to Europe as silent carriers [13,14]. In 2014, an epizootic occurred in Europe caused by a HPAI H5N8 clade 2.3.4.4c virus derived from the Gs/Gd lineage, which affected five poultry farms in the Netherlands (seven poultry farms in Europe) and caused limited mortality in wild birds [15–17]. This epizootic was succeeded by multiple epizootics caused by H5 clade 2.3.4.4b viruses [6,18]. In 2016, a HPAI H5N8 clade 2.3.4.4b virus caused outbreaks in eight poultry farms in the Netherlands (1134 poultry farms in Europe) and, in contrast to the viruses of H5 clade 2.3.4.4c, mass mortality in wild Anseriformes, especially tufted ducks *Aythya fuligula* and Eurasian wigeons *Anas Penelope* in Europe [19–23]. However, in 2017 a HPAI H5N6 clade 2.3.4.4b virus caused outbreaks in only three poultry farms in the Netherlands (five poultry farms in Europe), and wild bird mortality was limited [24–26].

In previous animal studies, significant differences in pathology of wild birds and poultry experimentally infected with HPAI H5 viruses isolated in the Netherlands during the epizootics of H5N8-2014, H5N8-2016 and H5N6-2017 were observed [14,18,27]. These three viruses all showed high pathogenicity in chickens [18]. However, Pekin ducks and Eurasian wigeons only showed mild disease after experimental infection with the H5N8-2014 and H5N8-2016 isolates, but severe pathogenicity was observed in both species after H5N6-2017 virus infection [14,18,28]. This underlines that pathogenicity in wild birds and poultry can differ between HPAI clade 2.3.4.4 viruses. In addition, wild bird mortality in nature may not directly link to the pathogenicity measured in animal experiments since wild bird mortality was limited during the H5N6-2017 epizootic, but pathogenicity was high during experimental infection in Eurasian wigeons.

In late 2020, a HPAI H5N1 clade 2.3.4.4b virus was detected in Europe but was quickly succeeded by several other genetically distinct HPAI H5N1 clade 2.3.4.4b viruses. In Europe, an additional nomenclature system was implemented using letters to further distinguish between H5N1 genotypes based on all eight viral genome segments, not to be confused with the clade system which is based solely on the Hemagglutinin segment. In the Netherlands, the HPAI H5N1 clade 2.3.4.4b genotype C first emerged in 2020 and caused a modest epizootic [29]. Surprisingly, wild bird mortality shifted from primarily tufted ducks and Eurasian wigeons during the previous H5 clade 2.3.4.4b epizootics (2016-2018) to Barnacle geese *Branta leucopsis* in 2020 in Europe [30,31]*.* Then, early 2021, a novel HPAI H5N1 virus (genotype AB) emerged which caused the largest epizootic in poultry and wild birds recorded in Europe to date (62 poultry farms in the Netherlands, 2296 poultry farms in Europe) [29]. Wild bird mortality was extremely high during the HPAI H5N1-2021-AB epizootic. Similar to the preceding epizootic caused by the H5N1-2020-C virus, Barnacle geese were the primarily affected species in Europe [32].

Limited information is available on the pathogenicity of the different HPAI H5N1 genotypes for wild bird species and poultry. Differences in pathogenicity can significantly influence virus transmission dynamics on farms and in wild bird populations and develop into an important factor determining the scale of an epizootic. Comprehensive data on virus shedding, mortality and morbidity in different poultry and wild bird species may reveal the impact of the recent HPAI H5N1-2020-genotype C and H5N1-2021-genotype AB viruses on these species and elucidate the potential role of species-specific pathogenicity in the extent of an epizootic. Importantly, pathogenicity after intravenous (same H5N8-2016 virus used in intra-respiratory infection study) and muscular inoculation (similar HPAI H5N8-2016 clade 2.3.4.4b) was high in Pekin ducks compared to intra-respiratory inoculation highlighting the need to study pathogenicity via the more natural respiratory inoculation route [28,33].

This study aimed to identify differences in pathogenicity of two recent HPAI H5 clade 2.3.4.4b viruses isolated in the Netherlands. Chickens (*Gallus gallus domesticus*), Pekin ducks (*Anas platyrhynchos domesticus*), Eurasian wigeons (*Mareca penelope*) and Barnacle geese (*Branta leucopsis*) were experimentally infected with HPAI H5N1 virus isolated in 2020 (genotype C) and HPAI H5N1 isolated in 2021 (genotype AB) to compare their pathogenicity.

## Methods

### Ethical statement

The animal experiment and procedures were in accordance with the national regulations on animal experimentation and the project license was approved by the Dutch Central Authority for Scientific procedures on Animals (CCD) (license number AVD40100202215972; experiment number 2021.D-0036.002) in the Netherlands. The animal procedures were performed conform the guidelines from the European Union directive 2010/63/EU.

### Virus preparation and phylogenetic analysis

A/Chicken/Netherlands/20019879-001005/2020 (H5N1-2020-C) and A/chicken/Netherlands/21037287-006010/2021 (H5N1-2021-AB) isolates, respectively EPI_ISL_17791407 and EPI_ISL_9856775, originate from index cases on poultry farms in the Netherlands. Viruses were passaged twice in 9- to 11-day-old specific pathogen free (SPF) embryonated chicken eggs (ECE, obtained from Royal GD, Deventer, the Netherlands). Whole genome sequencing was performed for both viruses on the originally isolated material and the second egg passage as previously described [34]. No mutations were observed between the seed material and E2 passages. Median egg infective dose (EID_50_) of the virus isolates were determined by end-point titration on 9- to 11-day-old SPF ECE’s. The virus isolates were titrated in triplicate on different days and EID50 titres were calculated using Reed and Muench [35].

Phylogenetic analysis of the complete genome sequences was performed for each genome segment separately, aligning the virus sequences using MAFFT v7.475 [36], reconstructing the phylogeny using maximum likelihood (ML) analysis with IQ-TREE software v2.0.3 and 1000 bootstrap replicates [37]. ML tree was visualized using the R package ggtree [38]. The GISAID sequences used in the phylogenetic analysis are listed in Supplementary table 2 [39].

### Animals and housing

Six-week-old SPF White Leghorn chickens were obtained from Royal GD (Deventer, the Netherlands) and six-week-old Pekin ducks were obtained from a commercial breeder farm. Since Eurasian wigeons and Barnacle geese are seasonal breeders, the age of the birds varied between 4 and 8 weeks. Both species were obtained from several hobby holdings in the Netherlands. All birds used in this study were from both sexes.

The experiment was performed in biosafety level 3 (BSL3) facilities at Wageningen Bioveterinary Research (WBVR, Lelystad, the Netherlands). Housing for Eurasian wigeons, Pekin ducks and chickens was arranged as previously described [18] and housing for Barnacle geese was identical to Eurasian wigeons and Pekin ducks. In short, optimal temperature (18.2–22.3°C), humidity and light conditions (14 h light, 10 h dark) were used. Eurasian wigeons, Barnacle geese and chickens were housed in one stable for each virus. The different species were separated with solid livestock panels. Pekin ducks inoculated with both viruses were housed in the same stable, separated by solid livestock panels and additional plastic sheets to prevent cross contamination of the virus. Feed and water were provided *ad libitum*. A mixture of straw and wood curls was used as bedding material for all bird species. Eurasian wigeons and Barnacle geese were treated preventively for coccidiosis by the addition of Baycox (Bayer, according to prescription) in drinking water for 48 h because these birds were obtained from several hobby holdings. Pekin ducks were also treated for coccidiosis to maintain equal conditions between experimental groups. After the Baycox treatment, swimming ponds were provided to the Barnacle geese, Eurasian wigeons and Pekin ducks.

### Experimental design

Birds were numbered randomly and allocated to an experimental group (no blinding of the experiment)(Table 1). Each experimental group contained 16 birds (two uninfected birds per group). To exclude previous avian influenza virus (AIV) infections in Barnacle geese, Eurasian wigeons and Pekin ducks blood samples, choana and cloaca swabs were taken on the day of arrival. Swabs were taken again after the seven-day acclimatization period to exclude ongoing AIV infections.

**Table 1.** Overview of the experiment.

Species	Virus	Histo-pathology 0 dpi^a^	IRPI	Histo-pathology 2 dpi^b^	Histo-pathology 3 dpi	Histo-pathology 10 dpi^c^
Chickens	H5N1-2020-C	2	10	4	–	–
	H5N1-2021-AB	2	10	4	–	–
Pekin ducks	H5N1-2020-C	2	10	–	4	4
	H5N1-2021-AB	2	10	–	4	4
Eurasian wigeons	H5N1-2020-C	2	10	–	4	4
	H5N1-2021-AB	2	10	–	4	4
Barnacle geese	H5N1-2020-C	2	10	–	4	4
	H5N1-2021-AB	2	10	–	4	4

Each group consisted of 16 birds: 10 birds for intra-respiratory pathogenicity index (IRPI), and 4 birds for necropsy 2 or 3 dpi. At the end of the experiment, 10 dpi, necropsy was performed at 4 birds of the IRPI birds. ^a^For each experimental group, two birds were used as negative controls (pre-inoculation). ^b^For chickens post mortem examination and tissue collection was scheduled at 2 dpi due to expected mortality after 48 h of infection. ^c^Birds from the IRPI group.

For challenge material, virus stocks were diluted in 2.95% tryptose phosphate buffer (TPB) to 10^6^ EID_50_/ml. Birds were intra-nasally (0.1 ml) and intra-choanal (0.1 ml) inoculated (total dosage of 10^5.3^ EID_50_ per bird) to ensure successful infection of the upper respiratory tract in all investigated species. For all four species, ten birds were inoculated per virus and monitored for ten days to determine the intra-respiratory pathogenicity index (IRPI) of the viruses according to the intravenous pathogenicity index (IVPI) scoring method [40]. Choana and cloaca swabs were collected daily from all birds and stored at −70°C until testing. Four birds per species were euthanized at 0 days post inoculation (dpi) to assess background pathology. After ten days, four birds with the lowest identification number from the IRPI experimental groups were examined by necropsy and tissues were collected for histopathology. Serum of the surviving birds was collected and stored at −20°C until testing. Additionally, to determine histopathology in the acute stage of the infection, four Barnacle geese, Eurasian wigeons and Pekin ducks were inoculated per virus and examined by necropsy at 3 days post inoculation (dpi). Since disease progression is faster in chickens, four birds were inoculated per virus, but were examined by necropsy on 2 dpi.

### Virus detection and antibody detection

AIV RNA from the swab material and standard curves was extracted using the MagNA Pure 96 system (Roche, Basel, Switzerland). AIV was detected by a quantitative real-time RT-PCR targeting the matrix gene (M-PCR), as previously described [34]. Standard curves were generated by making tenfold dilutions of the virus stocks in 2.95% TPB and subsequently freezing the log −2 to −9 dilutions at −70°C. Standard curves were used to calculate EID_50_ equivalents.

Antibodies against AIV nucleoprotein were detected using in house NP-ELISA as previously described [41]. Swabs and serum collected during the seven days acclimatization period were tested directly without freezing for presence of AIV RNA by M-PCR and AIV antibodies by NP-ELISA. Swabs and serum collected after inoculation of the birds was tested after freezing at −70 and −20°C respectively.

### Histopathology

Tissues of the respiratory tract (conchae, trachea, lung, air sac), digestive tract (ileum, colon and cloaca), central nervous system (brain, cerebrum) heart, liver and pancreas were collected of each bird selected for necropsy. The tissues were fixated for a minimum of 48 h and a maximum of one week in 10% neutral-buffered formalin, processed routinely and embedded in paraffin. Immunohistochemistry (IHC) to detect the presence of influenza A nucleoprotein and haematoxylin and eosin (HE) stain to evaluate histopathological changes were performed as previously described [27].

The IHC stain was semi-quantitative evaluated for the extension of viral expression and was graded by a board-certified veterinary pathologist: no staining (grade 0), sparse staining, focal or multifocal, < 5 foci in whole investigated tissue (grade 1), moderate staining, multifocal, > 5 foci (grade 2), abundant staining, multifocal to coalescing (grade 3) and excessive staining, almost diffuse staining (4). The HE stain was semi-quantitative evaluated for characteristics and severity of histopathologic changes and was scored subjectively by a board-certified veterinary pathologist on a scale of 0–3 with 0 indicating no influenza-associated lesion and 3 indicating severe influenza-related lesions.

### Statistics

Analysis was performed using the software package R (version 4.1.0) [42]. Mortality between the two HPAI H5N1 viruses within the same bird species was compared using the Kaplan–Meier (log-rank) test [43]. Shedding characteristics, defined as the AUC and shedding duration, were compared between the two HPAI H5N1 viruses within each bird species. After assessing normality (Shapiro test) comparisons for the mean AUC levels were done using the Kruskal–Wallis test [44]. Length of shedding was compared using the Kaplan-Meier (log-rank) test. The library “survival” was used for the survival analysis and the library “rstatix” was used for the remaining statistical test. The threshold for significance was set to *p < 0.05*.

## Results

### Genetic analysis of the two HPAI H5N1 viruses

Both HPAI H5N1 viruses belong to clade 2.3.4.4b and are highly related to wild bird sequences isolated during the subsequent epizootics. The largest genetic distance between the H5N1-2020-C and H5N1-2021-AB viruses was identified on PB2 (91.74% nucleotide similarity) followed by a smaller genetic distance on PA (97.26% nucleotide similarity) and minor genetic distances on the remaining six segments (98.25–99.42% nucleotide similarity) (Supplementary figure 1). Virus sequences were screened for previously identified virulence factors known to influence virulence, host specificity or binding of host proteins using Fluserver and FluMut [45,46]. Several amino acid changes in the PB2, PA, HA and NS1 segments were identified, but only four mutations that were previously identified as virulence factors, differed between the H5N1-2020-genotype C and H5N1-2021-genotype AB sequences. M1-101R (present in H5N1-2020-C, M1-101 K present in H5N1-2021-AB) in combination with M1-105R was shown to be more virulent in mice then M1-101S and M1-105S [47]. NS1-171 was identified as a binding site for the host protein phosphatidylinositol 3-kinase (PI3 K) (NS1-171D in H5N1-2020-C, NS1-171N in H5N1-2021-AB). NS2-67E in combination with NS2-32 T increases the amount of defective interfering particles (NS2-67E in H5N1-2020-C, NS2-67G in H5N1-2020-AB) [48]. Lastly, NP-M105 V (NP-105 V in H5N1-2021-C, NP105M in H5N1-2021-AB) was found to increase virulence in chickens [49].

### Mortality of the two HPAI H5N1 viruses in different bird species

All inoculated birds became infected and excreted influenza virus from both the choana and cloaca. All birds that survived the inoculation with either H5N1 virus tested positive for antibodies against influenza virus at the end of the experiment.

**Figure 1.**
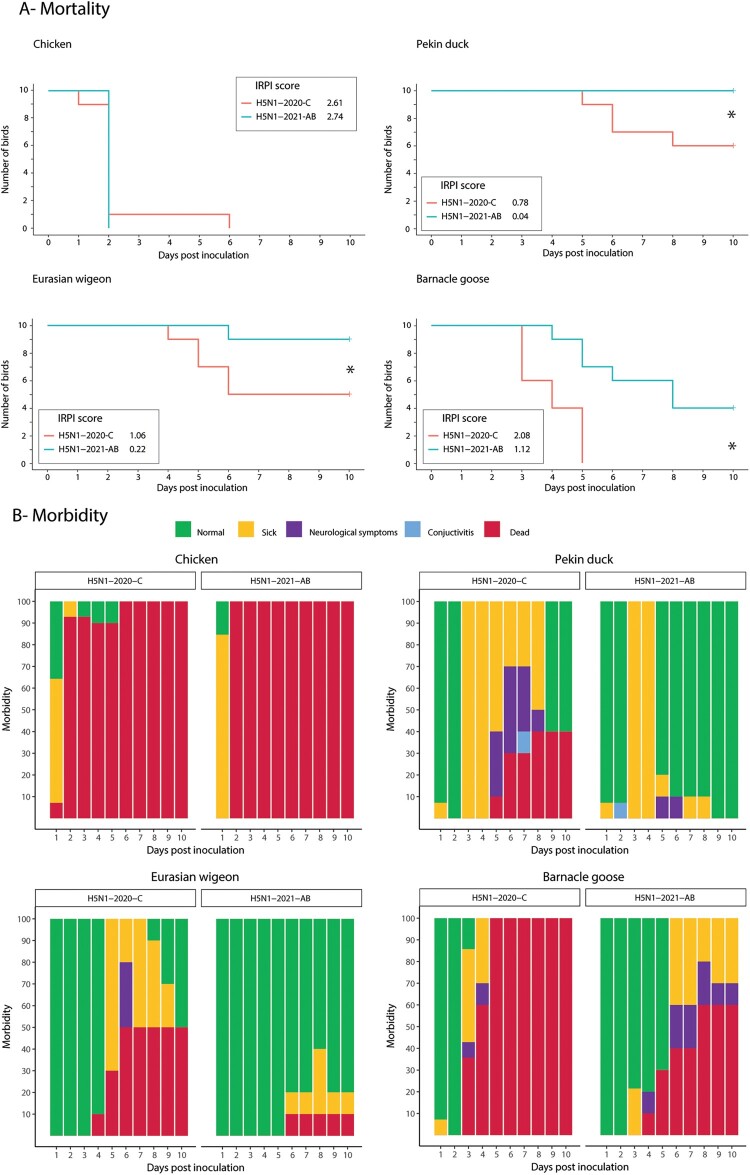
(a) Survival curves of chickens, Pekin ducks, Eurasian wigeons and Barnacle geese inoculated with HPAI H5N1-2020-C and H5N1-2021-AB. Ten birds were inoculated per experimental group and were monitored for clinical signs and mortality up to 10 dpi. Intra-respiratory pathogenicity indexes (IRPI) were calculated using the standard IVPI scoring method, where 3 is the maximum score. Significance is determined with a cut-off value of *p* < 0.05 (*). (b) Morbidity of all 14 birds/group. Normal (green) were apparently healthy birds. Sick (yellow) includes birds with reduced food intake, mild to severe lethargy and a hunched back. Neurological signs (purple) includes turning of the neck and head, tremors and paralysis. Conjunctivitis (light blue) and mortality (red) were also included.

For both viruses, all ten chickens in the IRPI group died within 2 dpi, except one in the H5N1-2020-C group that died at 6 dpi (Figure 1a). The calculated IRPI-score was high for both virus isolates: 2.61 for H5N1-2020-C and 2.74 for H5N1-2021-AB. No significant difference in mortality was found between the two H5N1 viruses.

In Pekin ducks and Eurasian wigeons, all ten birds survived after inoculation with H5N1-2021-AB except for one Eurasian wigeon on 6 dpi. The calculated IRPI score was low for both Pekin ducks (0.04) and Eurasian wigeons (0.22) (Figure 1a). Pekin ducks and Eurasian wigeons inoculated with H5N1-2020-C showed significantly higher mortality rates which was reflected in their calculated IRPI scores: Pekin ducks (0.78) and Eurasian wigeons (1.06).

Four out of ten Barnacle geese inoculated with H5N1-2021-AB survived the infection which resulted in a significantly lower calculated IRPI score of 1.12 (Figure 1a). None of the Barnacle geese infected with H5N1-2020-C survived the challenge and died within 3–5 dpi, which resulted in a significantly higher IRPI score of 2.08.

### Morbidity for different bird species infected with the two HPAI H5N1 viruses

Figure 1(b) shows the overall morbidity recorded for all 14 birds per group in this study. 9/14 chickens inoculated with H5N1-2020-C and 12/14 chickens inoculated with H5N1-2021-AB showed clinical signs at 1 dpi and no clear differences could be observed between the two H5N1 virus isolates. Lethargy and a hunched back were the most frequently observed clinical signs for both viruses in chickens. Excessive mucus formation from the nose was observed in two chickens inoculated with H5N1-2021-AB at 2 dpi.

In Pekin ducks, clinical signs, as lethargy and reduced food intake, were observed after 3 dpi for both viruses. Additionally, six Pekin ducks inoculated with H5N1-2020-C developed neurological signs (tremors and partial paralysis) between 5 and 8 dpi. Three of these ducks died or reached the humane endpoint (HEP) while the other three birds survived and showed no clinical signs at the end of the experiment. In the H5N1-2021-AB group, only one Pekin duck showed neurological signs but survived until the end of the experiment.

The Eurasian wigeons inoculated with H5N1-2021-C showed no clinical signs before 5 dpi, except for one animal that died on 4 dpi with no prior clinical signs. Lethargy was recorded from 5 to 9 dpi for all remaining Eurasian wigeons and neurological signs were observed in three Eurasian wigeons. Three other Eurasian wigeons died without preceding neurological signs, but lethargy was recorded prior to death for these birds. Morbidity and mortality were delayed and lower for Eurasian wigeons inoculated with H5N1-2021-AB. Only three birds showed mild clinical signs between 6 and 10 dpi and one bird died with no prior clinical signs.

All Barnacle geese inoculated with H5N1-2020-C died within 5 dpi. Severe lethargy and reduced food intake were recorded in all geese and two geese showed neurological signs prior to the HEP. In contrast, 9 out of 14 geese inoculated with the H5N1-2021-AB virus did not show any clinical signs before 6 dpi. Lethargy was recorded in eight geese and four geese showed neurological signs including turning of the neck and head, tremors and paralysis of which one goose showed mild neurological signs until the end of the experiment. Two geese were found dead without showing clinical signs. Two other geese died after observing severe lethargy.

### Virus shedding for different bird species infected with the two HPAI H5N1 viruses

**Figure 2.**
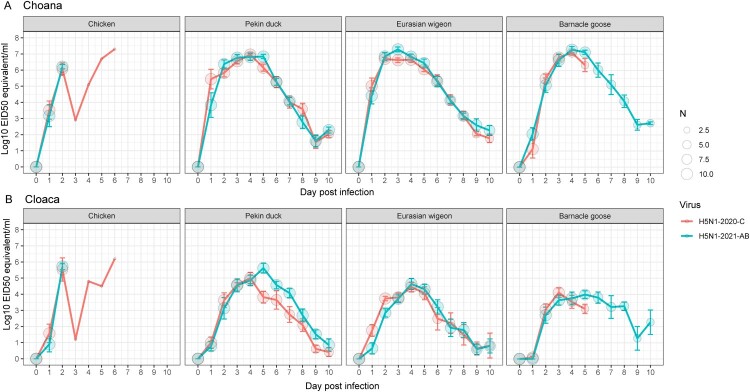
Virus shedding measured in choana and cloaca swabs. Average virus titre after inoculation with H5N1-2020-C (red) and H5N1-2021-AB (blue) virus in choana (a) and cloaca (b) swabs determined by real-time RT-PCR. The number of birds shedding virus (N) is depicted by the size of the circle and the error bars indicate the standard deviation.

The average virus shedding for the infected bird species was determined by a real-time RT-PCR and EID_50_ equivalents were calculated using standard curves. Total virus shedding (assessed by the AUC) and shedding duration was only determined for the ten birds in the IRPI group. Total virus shedding and duration were similar for both viruses and total virus shedding was lower in the cloaca swabs than in the choana swabs in all experimental groups (Figure 2 and Table 2). Respiratory shedding was lowest in chickens, but cloacal shedding was lowest in Barnacle geese. Significant differences in virus shedding (measured by the AUC) were observed between the two H5N1 viruses in Eurasian wigeons and Barnacle geese (Table 2). In addition, duration of shedding in Barnacle geese was significantly longer for the H5N1-2021-AB virus in both choana and cloaca swabs. Peak titre in both choana and cloaca swabs was at 2 dpi in chickens and 3–5 dpi for the Pekin ducks, Eurasian wigeons and Barnacle geese. Thus, virus shedding after infection with the H5N1-2020-C and H5N1-2021-AB viruses was highly similar in chickens, Pekin ducks, Eurasian wigeons and Barnacle geese but differences in virus shedding are observed between species.

**Table 2.** Shedding determined in choana and cloaca swabs by real-time RT-PCR.

Species	Virus	Choana swabs		Cloaca swabs	
		AUC	Duration	AUC	Duration
		Log10 EID50 equivalent titre ± SD	Days (LCL-UCL)	Log10 EID50 equivalent titre ± SD	Days (LCL-UCL)
Chicken	H5N1-2020-C	6.08 (0.77)	2.2 (1.2–3.2)	5.83 (0.64)	1.8 (1.14–2.46)
	H5N1-2021-AB	5.95 (0.39)	1.8 (1.5–2.1)	5.42 (0.63)	1.3 (0.95–1.65)
Pekin duck	H5N1-2020-C	7.39 (0.25)	8.4 (6.96–9.84)	5.57 (0.86)	7.2 (5.56–8.54)
	H5N1-2021-AB	7.57 (0.33)	9.5 (9.12–9.88)	6.17 (0.83)	8.4 (7.63–9.17)
Eurasian wigeon	H5N1-2020-C	7.29 (0.28)*	7.6 (5.75–9.45)	5.07 (0.66)	6.4 (5.00–7.8)
	H5N1-2021-AB	7.76 (0.32)*	9.4 (8.5–10.3)	5.15 (0.81)	7.1 (6.01–8.19)
Barnacle goose	H5N1-2020-C	7.17 (0.37)*	3.4 (2.9–3.9)*	4.37 (0.79)	3.1 (2.47–3.73)*
	H5N1-2021-AB	7.83 (0.36)*	7.4 (5.67–9.13)*	4.95 (0.53)	6.1 (4.65–7.55)*

The area under the curve (AUC) with standard deviation (SD) and duration of shedding with lower confidence limit (LCL) and upper confidence limit (UCL) was calculated for ten birds per experimental group over ten days (IRPI groups). Significance was determined with a cut-off value of *p* < 0.05 (*).

### Viral antigen expression and histopathology

**Figure 3.**
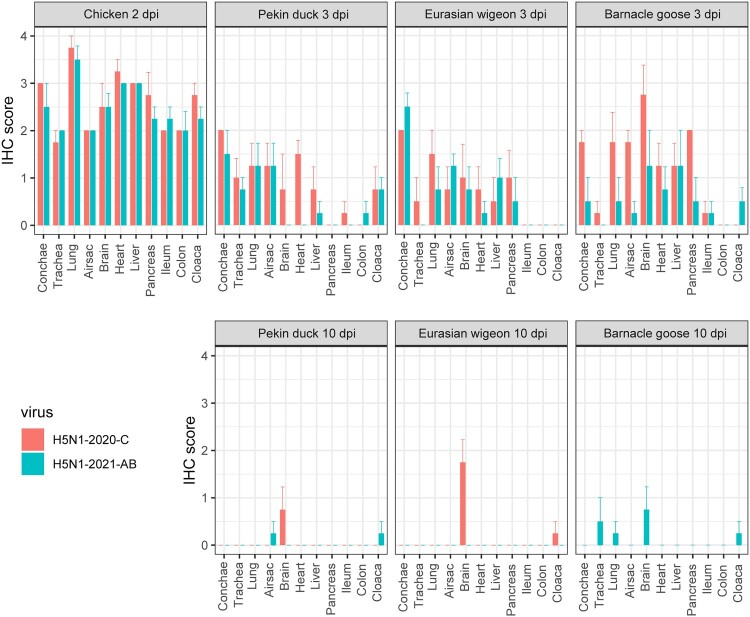
Average viral antigen expression (IHC) in collected tissues of chickens, Pekin ducks, Eurasian wigeons and Barnacle geese inoculated with H5N1-2020-C (red) or H5N1-2021-AB viruses (blue). Tissues were collected from four animals per species and virus at 2 dpi (chicken) or 3 and 10 dpi (only virus H5N1-2021-AB for Barnacle geese). Error bars indicate the standard deviation (SD).

**Figure 4.**
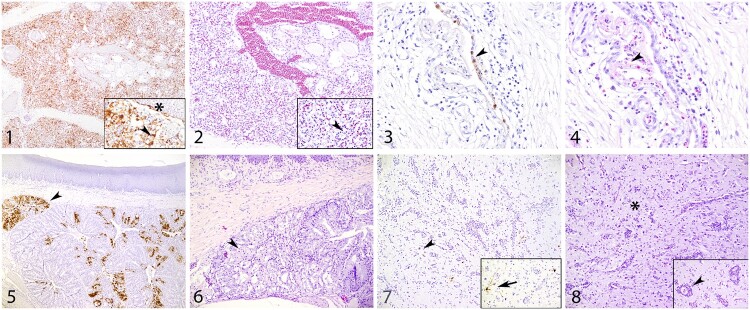
Viral antigen expression and histopathology. (1-2) Chicken lung (dpi 2), extensive viral staining in endothelial cells (asterisk), epithelial cells (pneumocytes) and mononuclear cells (arrowhead) (score 4) with associated fibronecrotic interstitial pneumonia (arrowhead) (score 3), 20x; (3-4) Pekin duck air sac (dpi 3), moderate staining of epithelial cells lining air sac (arrowhead) (score 2) with associate necrosis of epithelial cells with fibrin (arrowhead) (score 3), 40x; (5-6) Eurasian wigeon nasal conchae (dpi 3), multifocal staining of epithelial cells mucous glands (arrowhead) (score 3) with epithelial necrosis of mucous glands (arrowhead) (score 2), 10x and 20x; (7-8) Barnacle geese brain (cerebrum) (dpi 10), moderate viral staining of neurones (arrow) and glia cells (arrowhead) (score 2) with associated encephalomalacia, gliosis (asterisk) and perivascular cuffing (arrowhead)(score 3), 20x; (1, 3, 5 and 7) immunohistochemistry (IHC) influenza A nucleoprotein; (2, 4, 6 and 8) haematoxylin and eosin (HE).

*In situ* detection of AIV antigen and histopathology was performed in four birds during the acute phase of the infection (2 dpi for chickens and 3 dpi for other species) and for four birds at the end of the experiment (10 dpi). Overall, differences in tissue tropism were observed between bird species and viruses, but also in cell tropism (Supplementary table 1). No incidental lesions and minor background staining was observed after IHC of tissues from uninfected birds. Infected chickens showed no apparent difference in viral antigen expression between the two H5N1 viruses (Figure 3). Viral antigen expression was found in all investigated tissues for both virus isolates and endothelial virus expression was more prominent in chickens, compared to other bird species. The IHC score varied between 2 and 3, but the highest IHC score was noted in the lungs. Most severe histopathological changes were noted in the lungs by fibrinonecrotic interstitial pneumonia (Figure 4), in the brain large areas of encephalomalacia, in the liver varying amount of necrosis and loss or sinusoidal architecture, in the pancreas large areas of acinar necrosis, and in the alimentary tract extensive necrosis of lamina propria in ileum, colon, and cloaca.

In Pekin ducks the H5N1-2020-C virus was detected in respiratory organs, heart, liver, brain and to a lesser extent also in the digestive tract (ileum, cloaca) at 3 dpi. Brain lesions varied from rare perivascular cuffing (HE score 1) to areas of encephalomalacia in severe cases (HE score 3) at 3 dpi, which were often accompanied by neurological signs before necropsy. In contrast, the H5N1-2021-AB virus was exclusively detected in respiratory organs, the digestive tract and there was only limited expression in the liver, but the virus was not detected in the heart and brain at 3 dpi. The air sacs were the most severely affected part of the respiratory tract, especially at 3 dpi (epithelial degeneration and necrosis with large depositions of fibrin and necrotic debris Figure 4).

High amounts of viral antigen and associated necrosis of epithelial cells were detected in the mucous glands of the conchae (IHC score 2–3 Figure 4) of Eurasian wigeons inoculated with both H5N1 viruses at 3 dpi. Viral antigen was also detected to a lesser extent in other respiratory tissues (trachea, lung or air sac), however, no or less viral antigen was detected in the trachea and lung of Eurasian wigeons inoculated with H5N1-2021-AB at 3 dpi. Both viruses were also detected to a similar level in the brain and internal organs (heart, liver and pancreas) at 3 dpi, but no viral antigen was detected in the digestive tract for either of the H5N1 viruses. At 10 dpi overall histopathology was less severe in the Eurasian wigeons compared to 3 dpi and no virus antigen could be detected in any of the tissues collected in Eurasian wigeons inoculated with H5N1-2021-AB virus at 10 dpi, which indicates recovery for these birds. In contrast, Eurasian wigeons inoculated with H5N1-2020-C showed severe brain lesions at 10 dpi, indicated by areas of encephalomalacia and coinciding with neurological signs before necropsy.

Barnacle geese inoculated with H5N1-2020-C virus showed a similar pattern of viral expression as observed in the other Anseriformes species (Pekin duck and Eurasian wigeon). This expression was found in all respiratory organs, except for the trachea, but also in heart, liver and pancreas, but there was only limited expression in intestinal tract (only a few mononuclear cells stained positive). The most extensive viral expression was found in the neurones in the brain (score 2–4) at 3 dpi, however neuronal necrosis was limited at this time-point. None of the H5N1-2020-C infected Barnacle geese survived until 10 dpi. Compared to the H5N1-2020-C virus, Barnacle geese inoculated with H5N1-2021-AB virus expressed less viral antigen in the respiratory organs, heart, pancreas and brain at 3 dpi and at this time-point, viral antigen expression in brain, liver, heart and pancreas, was more prominent than in the respiratory and intestinal tract. At 10 dpi, only minimal to moderate viral expression was detected in the brain; however, 3 out of 4 birds showed moderate to severe encephalomalacia with gliosis and perivascular cuffing (Figure 4).

## Discussion

Multiple HPAI H5 clade 2.3.4.4b viruses have caused epizootics affecting wild birds and poultry between the years 2016–2023 in Europe. However, the number and species of wild birds that were found dead and the number of outbreaks on poultry farms varied greatly between the different epizootics. Interestingly, the HPAI H5N1-2020-genotype C virus did not cause a large epizootic, whereas the HPAI H5N1-2021-genotype AB caused the largest avian influenza epizootic recorded in Europe to date [29]. Wild bird surveillance during the H5 clade 2.3.4.4b epizootics between 2016 and 2018 recorded mostly deceased Tufted ducks and Eurasian wigeons in Europe [16,23,24]. However, dead wild bird surveillance during the 2020–2022 period in Europe showed that the most affected species during this epizootic were Barnacle geese, whereas less mortality amongst Tufted ducks and Eurasian wigeons was observed [20,30–32]. It is currently unclear which factors have influenced the differences in mortality of specific wild bird species during these epizootics, and how this has influenced the number of outbreaks in poultry. Wild bird dynamics, pre-existing immunity in wild bird populations, viral dynamics and virus characteristics may underlie these differences. Here we investigated pathogenicity and virus shedding of two HPAI H5N1 clade 2.3.4.4b viruses causing recent epizootics, by experimental infection of chickens, Pekin ducks, Eurasian wigeons and Barnacle geese.

The IRPI score in Pekin ducks (0.04) and Eurasian wigeons (0.22) inoculated with H5N1-2021-AB was very low, morbidity was short and mild, while virus shedding remained high. Interestingly, Pekin ducks and Eurasian wigeons inoculated with H5N1-2021-AB virus showed abundant viral antigen expression at 3 dpi with virus-induced necrosis of mainly epithelial and mononuclear cells in the nasal conchae, air sac, but also in the liver and pancreas (only Eurasian wigeons). At 10 dpi, the tissue lesion was less severe than at 3 dpi in both species, suggesting recovery from infection. This shows that the H5N1-2021-AB virus is able to replicate in various tissues of Pekin ducks and Eurasian wigeons without causing overt clinical signs. This may increase the risk of virus transmission by Eurasian wigeons over long distances compared to the H5N1-2020-C virus (transmission challenge not performed in this study). A comparison of the observed clinical signs on poultry farms in the Netherlands during the 2014–2018 and 2020–2022 seasons indicated a reduction in neurological, gastrointestinal and respiratory tract signs in Pekin ducks [50]. Therefore, the HPAI H5N1-2021-AB virus may cause subclinical infections on Pekin duck farms which increases the risk for spillback to wild birds, spillover to other farms, mammals and humans.

Neurological signs were observed in Pekin ducks, Eurasian wigeons and Barnacle geese inoculated with the H5N1-2020-C virus which aligns with detection of virus antigen and encephalomalacia in the brain at 3 and 10 dpi (Barnacle geese not analysed since no birds survived up to 10 dpi). In contrast, no viral antigen expression was detected in the brain of Pekin ducks at 3 and 10 dpi and Eurasian wigeons at 10 dpi which may explain the low mortality and morbidity observed in the Pekin ducks and Eurasian wigeons inoculated with H5N1-2021-AB virus.

Mortality in Barnacle geese was remarkably high for both HPAI H5N1 viruses, but total virus shedding and shedding duration for H5N1-2021-AB virus was comparable to the other Anseriformes species tested. Interestingly, viral antigen and virus-associated lesions were still detected at 10 dpi in lung, brain and cloaca of Barnacle geese. Coincidingly, clinical signs and virus shedding were still recorded for all remaining Barnacle geese at 10 dpi. This is in contrast with the Eurasian wigeons inoculated with H5N1-2021-AB which showed low morbidity and mortality and no sustained viral antigen expression. Therefore, the increased number of dead Barnacle geese found in nature during the 2020–2022 season may be the result of the high mortality caused by the recent HPAI H5N1 viruses. During the European 2021–2022 epizootic an unusual high number of mammals were found to be infected with HPAI H5N1, most likely as a result of scavenging from infected wild birds [51,52]. Barnacle geese are foraging on grasslands while Eurasian wigeons are located more frequently on water which may increase the likelihood that barnacle geese carcasses are consumed by carnivorous mammals. It remains to be determined whether the increased number of infections in mammals is caused by unknown mammalian adaptions or by the increased number of infected wild Barnacle geese, which is in line with the increased mortality observed in our study for Barnacle geese.

Our previous study showed that IRPI scores were low in Pekin ducks and Eurasian wigeons inoculated with an HPAI H5N8 clade 2.3.4.4b virus isolated in 2016, but that IRPI scores were high in both species after inoculation with a HPAI H5N6 clade 2.3.4.4b virus isolated in 2017 [18]. Coincidentally, both the H5N6-2017 and H5N1-2020-C viruses did not cause a large epizootic unlike the H5N8-2016 and H5N1-2021-AB viruses that both caused large epizootics [20,25,29]. Although transmission has not been determined in this study, lower pathogenicity (IRPI scores) combined with high virus shedding, particularly in wild birds, is likely to increase transmission efficiency and subsequently affect the scale of the epizootic.

Phylogenetic analysis of these two HPAI H5N1 viruses indicated that the largest genetic difference was present in the PB2 and PA segments, although differences were also observed on the remaining six gene segments. Three amino acid positions (M1-101R, NS1-171, NS2-67E)[47,48] were identified in mammals and one amino acid position (NP-M105 V)[49] was identified in chickens and were all present in the H5N1-2021-C sequence but not in the H5N1-2021-AB sequence. Further study will be required to determine if one or multiple of these previously identified virulence factors may cause the observed difference in pathogenicity between the two studied H5N1 viruses. For example, reverse genetics may be used to exchange gene segments and generate viruses with specific amino acid changes to reveal the genetic changes required for the measured difference in pathogenicity and possibly lead to the identification of the mechanism behind the difference in pathogenicity.

This study has several limitations. Although phylogenetic analysis indicated the selected viruses are similar to other H5N1 genotype C and AB viruses, pathogenicity measured for the viruses used in this study may not be representative for the pathogenicity of other genotype C and AB viruses. Furthermore, two wild bird species and two poultry species were selected as model species but our findings may not be translatable to other wild bird or poultry species. Transmission challenge studies could be helpful to determine if the lower pathogenicity of the HPAI H5N1-2021-AB virus for both Barnacle geese and Eurasian wigeons may extend to other wild bird species in the Anseriformes order which may have contributed to efficient spread of the virus amongst migrating wild birds and introductions into poultry causing the largest recorded avian influenza epizootic in Europe to date.

Overall, our study showed that pathogenicity in Pekin ducks, Eurasian wigeons and Barnacle geese after inoculation with H5N1-2021-AB virus was lower compared to the H5N1-2020-C virus. However, the virus shedding levels were similar for both viruses. Subclinical infections occurred in Pekin ducks and Eurasian wigeons infected with H5N1-2021-AB virus, whereas mortality for Barnacle geese was 100% for the H5N1-2020-C virus and 60% for the H5N1-2021-AB virus. The genetic factors that underlie this difference in pathogenicity remain to be determined, but may be involved in the more extensive neurotropism of the H5N1-2020-C virus or alterations in the immune response (not evaluated here).

Besides the HPAI H5N1 clade 2.3.4.4b genotype C and genotype AB viruses eighteen HPAI H5N1 clade 2.3.4.4b genotypes have emerged since the introduction in Europe in 2020 which likely continued to alter host range and virulence in different species [53]. For example, mass mortality was recorded in Charadriiformes (Skua, Gulls, Sandwich Terns) infected with HPAI H5N1 clade 2.3.4.4b genotype BB [54,55], carnivorous wild mammals [51,52] and domestic mammals [56–58] were found to be infected with HPAI H5N1 clade 2.3.4.4b. Even ruminants, which were previously not considered a host for HPAI viruses, were found to be infected by a HPAI H5N1 clade 2.3.4.4b virus from the North-American lineage [59] and bovine respiratory cells have appeared to be permissive to infection with European HPAI H5N1 clade 2.3.4.4b viruses [60]. Therefore, repeated evaluation of HPAI virus traits is required to determine the host range and virulence. This may lead to the identification of virulence factors which may help assess the risk of HPAI introductions in different species during future epizootics.

## Supplementary Material

Supplementary.pdf
