# Supplementary material for: Experimental infection of chickens, Pekin ducks, Eurasian wigeons and Barnacle geese with two recent highly pathogenic avian influenza H5N1 clade 2.3.4.4b viruses
Source: Emerg Microbes Infect. 2024 Sep 2;13(1):2399970. doi: 10.1080/22221751.2024.2399970 (PMC11395873; doi:10.1080/22221751.2024.2399970)
Supplement: Supplementary.pdf [file TEMI_A_2399970_SM5507.pdf]

Supplementary figure 1. Phylogenetic tree of the complete genome sequences with the strains used in this study (red), a random set of bird isolates from the 2020-2021 epizootic (purple), 2021-2022 epizootic (blue) and 2022-2023 epizootic (green) inferred using maximum likelihood methods. Genome sequences were obtained from the GISAID database (Supplementary table 2).

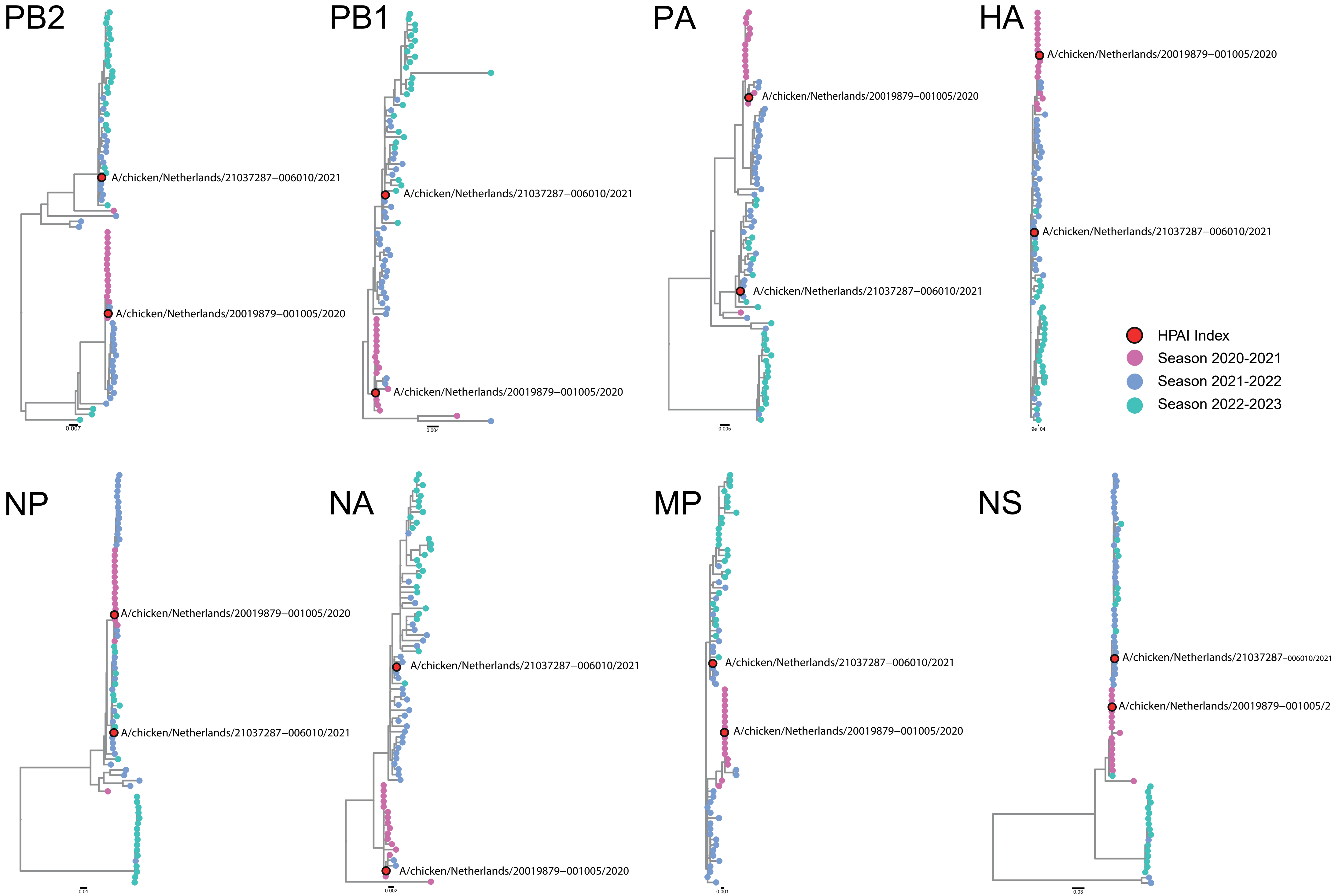

Supplementary table 1. Type of cells stained by immunohistochemistry (IHC). First mentioned cell type is most prominent.

**Organ**	**Species**			
	**Chicken**	**Pekin duck**	**Eurasian Wigeon**	**Barnacle Geese**
**Nose (conchae)**	endothelial cells of the submucosa and epithelial cells (mucus glands)	epithelial cells (mucus glands)	epithelial cells (mucus glands) and some mononuclear cells	epithelial cells (mucus glands) and some mononuclear cells
**Trachea**	endothelial cells and epithelial cells	epithelial cells	epithelial cells	mononuclear cells in submucosa and vascular cavities of the cartilage, few endothelial cells
**Lung**	endothelial cells and epithelial cells (pneumocytes) and mononuclear cells	epithelial cells (pneumocytes)	epithelial cells (pneumocytes and bronchi) and mononuclar cells	epithelial cells (pneumocytes) and mononuclear cells
**Air sac**	endothelial cells and epithelial cells (cells lining air sac)	epithelial cells (cells lining air sac) and mononuclear cells	mononuclear cells and epithelial cells (cells lining air sac)	mononuclear cells and epithelial cells (cells lining air sac)
**Heart**	cardiomyocytes and endothelial cells	cardiomyocytes	cardiomyocytes	cardiomyocytes
**Liver**	endothelial cells and mononuclear cells (Kupfer cells), few hepatocytes.	endothelial cells, few hepatocytes	mononuclear cells, perhaps few hepatocyes	mononuclear cells, perhaps few hepatocyes
**Pancreas**	epithelial cells (acinar cells)	no staining in pancreas	epithelial cells	epithelial cells
**Ileum**	mononuclear cells and endothelial cells and myocytes of the lamina muscularis	endothelial cell	no staining	a few mononuclear cells in lamina propria/follicles
**Colon**	mononuclear cells and endothelial cells and myocytes of the lamina muscularis	mononuclear cell	no staining	no staining
**Cloaca**	endothelial cells and mononuclear cells	epithelial cells (mucosa)	a few mononuclear cells	a few mononuclear cells
**Brain (cerebrum)**	neurons and endothelial cells and epithelial cells lining ventricles (ependymal cells)	epithelial cells lining ventricles (ependymal cells) and neurons	mostly neurons and lesser glia cells	mostly neurons and lesser glia cells and epithelial cells lining ventricles (ependymal cells)

Supplementary table 2. GISAID accession numbers.

We gratefully acknowledge the authors, originating and submitting laboratories of the sequences from GISAID's EpiFlu™ Database on which this research is based. The list is detailed below.
All submitters of data may be contacted directly via www.gisaid.org

Isolate-ID	Country	Collection date	Isolate name	Originating Lab	Submitting Lab	Authors
EPI_ISL_18048779	Czech Repub	2023-Jul-17	A/peregrine falcon/Czech Republic/10854/2023	State Veterinary Institute Prague	State Veterinary Institute Prague	Alexander,Nagy;Lenka,Cernikova;Martina,Stara
EPI_ISL_9603901	Czech Repub	2021-Nov-18	A/goose/Czech Republic/22608-3T/2021	State Veterinary Institute Prague	State Veterinary Institute Prague	Alexander,Nagy;Lenka,Cernikova;Martina,Stara
EPI_ISL_956412	Italy	2020-Nov-23	A/greater_white-fronted_goose/Italy/20VIR8073-4/2020	Istituto Zooprofilattico Sperimentale delle Venezie, EU/OIE/Reference Laboratory and FAO Reference Centre for AI and ND	Istituto Zooprofilattico Sperimentale Delle Venezie	Zecchin, B.; Fusaro, A.; Milani, A.; Schivo, A.; Salviato, A.; Pastori, A.; Zamperin, G.; Monne, I.; Terregino, C.
EPI_ISL_14864637	United Kingdo	2022-Jun-16	A/goose/England/317610/2022	Animal and Plant Health Agency (APHA)	Animal and Plant Health Agency (APHA)	
EPI_ISL_14576106	Netherlands	2022-Aug-11	A/greylag goose/Netherlands/22013818-001/2022	Wageningen Bioveterinary Research	Wageningen Bioveterinary Research	Beerens, Nancy; Harders, Frank; Pritz-Verschuren, Sylvia; Roose, Marit; Venema, Sandra; Germeraad, Evelien; Engelsma, Marc; Heutink, Rene
EPI_ISL_7952126	Netherlands	2021-Nov-07	A/greylag goose/Netherlands/21038417-001/2021	Wageningen Bioveterinary Research	Wageningen Bioveterinary Research	Beerens, Nancy; Harders, Frank; Pritz-Verschuren, Sylvia; Roose, Marit; Venema, Sandra; Germeraad, Evelien; Engelsma, Marc; Heutink, Rene
EPI_ISL_7267243	Netherlands	2021-Nov-09	A/greylag goose/Netherlands/21038570-002/2021	Wageningen Bioveterinary Research	Wageningen Bioveterinary Research	Beerens, Nancy; Harders, Frank; Pritz-Verschuren, Sylvia; Roose, Marit; Venema, Sandra; Germeraad, Evelien; Engelsma, Marc; Heutink, Rene
EPI_ISL_17514171	Austria	2023-Jan-20	A/black-headed gull/Austria/23006953/2023	Institute for Veterinary Disease Control Moedling, Austrian Agency for Health and Food Safety	Austrian Agency for Health and Food Safety (AGES)	
EPI_ISL_17679759	Italy	2023-Feb-18	A/black-headed_gull/Italy/23VIR1484-4/2023	Istituto Zooprofilattico Sperimentale delle Venezie, EU/OIE/Reference Laboratory and FAO Reference Centre for AI and ND	Istituto Zooprofilattico Sperimentale Delle Venezie	Pastori, A.; Zecchin, B.; Fusaro, A.; Schivo, A.; Salviato, A.; Palumbo, E.; Giussani, E.; Monne, I.; Terregino, C.
EPI_ISL_18043920	Belgium	2023-Jan-24	A/Larus_argentatus/Belgium/00906_0001/2023	Sciensano - Animal Infectious Diseases	Sciensano, Department of Animal Infectious Diseases	Van Borm, Steven; Roupie, Virginie; Hostyn, Pierre; Mathijs, Elisabeth; Lambrecht, Benedicte; Steensels, Mieke
EPI_ISL_14389133	Belgium	2022-Jan-06	A/Larus_argentatus/Belgium/595_0008/2022	Sciensano - Animal Infectious Diseases	Sciensano, Department of Animal Infectious Diseases	Van Borm, Steven; Vandenbussche, Frank; Roupie, Virginie; Lambrecht, Benedicte; Steensels, Mieke
EPI_ISL_18373694	Netherlands	2023-Aug-25	A/European Herring Gull/Netherlands/23013704-002/2023	Wageningen Bioveterinary Research	Wageningen Bioveterinary Research	Beerens, Nancy; Harders, Frank; Pritz-Verschuren, Sylvia; Roose, Marit; Venema, Sandra; Germeraad, Evelien; Engelsma, Marc; Heutink, Rene
EPI_ISL_18983379	Spain	2023-Mar-07	A/Larus ridibundus/Spain/CR4063/2023	Centro de Investigación en Sanidad Animal (CReSA)	Hospital Universitari Vall d'Hebron	Andres, C.; Gonzalez-Sanchez, A; Pinana, M; Prats, I; Martin, MC.; Garcia-Comunas, K; Piquer, M; Pumarola, T; Anton, A.
EPI_ISL_13617698	Netherlands	2022-Jun-13	A/sandwich tern/Netherlands/22010742-002/2022	Wageningen Bioveterinary Research	Wageningen Bioveterinary Research	Beerens, Nancy; Harders, Frank; Pritz-Verschuren, Sylvia; Roose, Marit; Venema, Sandra; Germeraad, Evelien; Engelsma, Marc; Heutink, Rene
EPI_ISL_17679782	Italy	2023-Jan-19	A/swan/Italy/23VIR682-2/2023	Istituto Zooprofilattico Sperimentale delle Venezie, EU/OIE/Reference Laboratory and FAO Reference Centre for AI and ND	Istituto Zooprofilattico Sperimentale Delle Venezie	Pastori, A.; Zecchin, B.; Fusaro, A.; Schivo, A.; Salviato, A.; Palumbo, E.; Giussani, E.; Monne, I.; Terregino, C.
EPI_ISL_766876	United Kingdo	2020-Dec-03	A/mute_swan/England/234255/2020	Animal and Plant Health Agency (APHA)	Animal and Plant Health Agency (APHA)	
EPI_ISL_1139102	Netherlands	2020-Nov-10	A/swan/Netherlands/20017772-002/2020	Wageningen Bioveterinary Research	Wageningen Bioveterinary Research	Beerens, Nancy; Harders, Frank; Pritz-Verschuren, Sylvia; Roose, Marit; Germeraad, Evelien; Engelsma, Marc; Bossers, Alex; Heutink, Rene
EPI_ISL_1139101	Netherlands	2020-Nov-09	A/swan/Netherlands/20017605-002/2020	Wageningen Bioveterinary Research	Wageningen Bioveterinary Research	Beerens, Nancy; Harders, Frank; Pritz-Verschuren, Sylvia; Roose, Marit; Germeraad, Evelien; Engelsma, Marc; Bossers, Alex; Heutink, Rene
EPI_ISL_17212547	United Kingdo	2020-Dec-03	A/mute swan/England/SA14-234255/2020	Animal and Plant Health Agency (APHA)	Animal and Plant Health Agency (APHA)	Seekings, AH
EPI_ISL_7952115	Netherlands	2021-Nov-24	A/mute swan/Netherlands/21039526-002/2021	Wageningen Bioveterinary Research	Wageningen Bioveterinary Research	Beerens, Nancy; Harders, Frank; Pritz-Verschuren, Sylvia; Roose, Marit; Venema, Sandra; Germeraad, Evelien; Engelsma, Marc; Heutink, Rene
EPI_ISL_15857846	Italy	2022-Nov-02	A/turkey/Italy/22VIR10627-4/2022	Istituto Zooprofilattico Sperimentale delle Venezie, EU/OIE/Reference Laboratory and FAO Reference Centre for AI and ND	Istituto Zooprofilattico Sperimentale delle Venezie	Barbierato, G.; Zecchin, B.; Fusaro, A.; Schivo, A.; Salviato, A.; Palumbo, E.; Giussani, E.; Pastori, A.; Monne, I.; Terregino, C.

EPI_ISL_14760747	Italy	2021-Dec-15	A/turkey/Italy/21VIR11251-1/2021	Istituto Zooprofilattico Sperimentale delle Venezie, EU/OIE/Reference Laboratory and FAO Reference Centre for AI and ND	Istituto Zooprofilattico Sperimentale delle Venezie	Barbierato, G.; Zecchin, B.; Fusaro, A.; Schivo, A.; Salviato, A.; Palumbo, E.; Giussani, E.; Pastori, A.; Monne, I.; Terregino, C.
EPI_ISL_14760660	Italy	2021-Nov-30	A/turkey/Italy/21VIR10471/2021	Istituto Zooprofilattico Sperimentale delle Venezie, EU/OIE/Reference Laboratory and FAO Reference Centre for AI and ND	Istituto Zooprofilattico Sperimentale delle Venezie	Barbierato, G.; Zecchin, B.; Fusaro, A.; Schivo, A.; Salviato, A.; Palumbo, E.; Giussani, E.; Pastori, A.; Monne, I.; Terregino, C.
EPI_ISL_15234363	Spain	2022-Aug-04	A/turkey/Spain/2755-6_22VIR8632-2/2022	Laboratorio Central de Veterinaria	Istituto Zooprofilattico Sperimentale delle Venezie	Ruano, M.J.; Rocha, A.; Sanchez, A.; Aguero, M; Barbierato, G.; Zecchin, B.; Fusaro, A.; Schivo, A.; Salviato, A.; Palumbo, E.; Pastori, A.; Giussani, E.; Monne, I.; Terregino, C.
EPI_ISL_11259300	Spain	2022-Feb-08	A/turkey/Spain/490-24_22VIR2142-27/2022	Laboratorio Central de Veterinaria	Istituto Zooprofilattico Sperimentale delle Venezie	Ruano, M.J.; Rocha, A.; Sanchez, A.; Aguero, M; Barbierato, G.; Zecchin, B.; Fusaro, A.; Schivo, A.; Salviato, A.; Palumbo, E.; Giussani, E.; Monne, I.; Terregino, C.
EPI_ISL_5146481	Germany	2021-May-03	A/turkey/Germany-NI/AI04425/2021	Lebensmittel- und Veterinärinstitut Oldenburg - Standort Veterinärinstitut	Friedrich-Loeffler-Institut	
EPI_ISL_15234355	Spain	2022-Jun-29	A/red-backed-hawk/Spain/2313-1_22VIR8632-10/2022	Laboratorio Central de Veterinaria	Istituto Zooprofilattico Sperimentale delle Venezie	Ruano, M.J.; Rocha, A.; Sanchez, A.; Aguero, M; Barbierato, G.; Zecchin, B.; Fusaro, A.; Schivo, A.; Salviato, A.; Palumbo, E.; Pastori, A.; Giussani, E.; Monne, I.; Terregino, C.
EPI_ISL_17996130	Italy	2023-Jun-16	A/sandwich_tern/Italy/23VIR5828-2/2023	Istituto Zooprofilattico Sperimentale delle Venezie, EU/OIE/Reference Laboratory and FAO Reference Centre for AI and ND	Istituto Zooprofilattico Sperimentale Delle Venezie	Pastori, A.; Zecchin, B.; Fusaro, A.; Schivo, A.; Salviato, A.; Palumbo, E.; Giussani, E.; Monne, I.; Terregino, C.
EPI_ISL_18538403	United Kingdo	2023-Sep-26	A/pheasant/England/121396/2023	Animal and Plant Health Agency (APHA)	Animal and Plant Health Agency (APHA)	
EPI_ISL_18258049	United Kingdo	2023-Aug-17	A/Pheasant/Scotland/107872/2023	Animal and Plant Health Agency (APHA)	Animal and Plant Health Agency (APHA)	
EPI_ISL_18209361	United Kingdo	2023-Aug-07	A/pheasant/Scotland/103986/2023	Animal and Plant Health Agency (APHA)	Animal and Plant Health Agency (APHA)	
EPI_ISL_16384165	United Kingdo	2022-Nov-24	A/pink-footed_goose/Scotland/163839/2022	Animal and Plant Health Agency (APHA)	Animal and Plant Health Agency (APHA)	
EPI_ISL_18445727	France	2022-Jun-07	A/Curlew/France/22P018028/2022	ANSES Agence Nationale De Securite Sanitaire De L'alimentation	ANSES Agence Nationale De Securite Sanitaire De L'alimentation	
EPI_ISL_18075148	Netherlands	2023-Mar-10	A/Peregrine Falcon/Netherlands/13/2023	Erasmus Medical Center	Erasmus Medical Center	Vuong,O; Thewessen, S; Bellido-Martin, A.B.; Fouchier, R.A.M.
EPI_ISL_17821061	Netherlands	2023-Jun-02	A/Black-headed Gull/Netherlands/76/2023	Erasmus Medical Center	Erasmus Medical Center	
EPI_ISL_17780483	Netherlands	2023-May-19	A/Common Tern/Netherlands/1/2023	Erasmus Medical Center	Erasmus Medical Center	Vuong,O;Thewessen, S; Bellido-Martin, A.B.;Fouchier, R.A.M.
EPI_ISL_15581789	Netherlands	2022-Oct-12	A/Mallard/Netherlands/19/2022	Erasmus Medical Center	Erasmus Medical Center	
EPI_ISL_15364797	Netherlands	2022-Sep-19	A/Mute Swan/Netherlands/2/2022	Erasmus Medical Center	Erasmus Medical Center	
EPI_ISL_15088302	Netherlands	2022-Aug-27	A/Greylag Goose/Netherlands/13/2022	Erasmus Medical Center	Erasmus Medical Center	
EPI_ISL_603135	Netherlands	2020-Oct-16	A/Eurasian Wigeon/Netherlands/5/2020	Erasmus Medical Center	Erasmus Medical Center	
EPI_ISL_603134	Netherlands	2020-Oct-16	A/Eurasian Wigeon/Netherlands/4/2020	Erasmus Medical Center	Erasmus Medical Center	
EPI_ISL_603133	Netherlands	2020-Oct-16	A/Eurasian Wigeon/Netherlands/1/2020	Erasmus Medical Center	Erasmus Medical Center	
EPI_ISL_7753443	Germany	2021-Oct-26	A/buzzard/Germany-SH/AI06210/2021	Landeslabor Schleswig-Holstein	Friedrich-Loeffler-Institut	
EPI_ISL_15857990	Italy	2022-Oct-17	A/Chicken/Italy/22VIR9832-3/2022	Istituto Zooprofilattico Sperimentale delle Venezie, EU/OIE/Reference Laboratory and FAO Reference Centre for AI and ND	Istituto Zooprofilattico Sperimentale delle Venezie	Barbierato, G.; Zecchin, B.; Fusaro, A.; Schivo, A.; Salviato, A.; Palumbo, E.; Giussani, E.; Pastori, A.; Monne, I.; Terregino, C.
EPI_ISL_14761041	Italy	2021-Dec-22	A/broiler/Italy/21VIR11583-1/2021	Istituto Zooprofilattico Sperimentale delle Venezie, EU/OIE/Reference Laboratory and FAO Reference Centre for AI and ND	Istituto Zooprofilattico Sperimentale delle Venezie	Barbierato, G.; Zecchin, B.; Fusaro, A.; Schivo, A.; Salviato, A.; Palumbo, E.; Giussani, E.; Pastori, A.; Monne, I.; Terregino, C.
EPI_ISL_11922809	Poland	2022-Feb-18	A/chicken/Poland/H157_22VIR2515-3/2022	National Veterinary Research Institute	Istituto Zooprofilattico Sperimentale delle Venezie	Swieton, E.; Smietanka, K.; Barbierato, G.; Zecchin, B.; Fusaro, A.; Schivo, A.; Salviato, A.; Palumbo, E.; Giussani, E.; Monne, I.; Terregino, C.
EPI_ISL_16997293	Czech Repub	2022-Dec-09	A/chicken/Czech_Republic/22968_orig/2022	State Veterinary Institute Prague	State Veterinary Institute Prague	Alexander,Nagy; Lenka,Cernikova; Martina,Stara
EPI_ISL_18378729	United Kingdo	2023-Aug-20	A/Chicken/Scotland/109635/2023	Animal and Plant Health Agency (APHA)	Animal and Plant Health Agency (APHA)	
EPI_ISL_17072779	United Kingdo	2023-Jan-27	A/Chicken/Scotland/015627/2023	Animal and Plant Health Agency (APHA)	Animal and Plant Health Agency (APHA)	
EPI_ISL_15585857	United Kingdo	2022-Sep-18	A/chicken/England/118935/2022	Animal and Plant Health Agency (APHA)	Animal and Plant Health Agency (APHA)	
EPI_ISL_1122425	United Kingdo	2020-Dec-15	A/chicken/England/043315/2020	Animal and Plant Health Agency (APHA)	Animal and Plant Health Agency (APHA)	
EPI_ISL_17791407	Netherlands	2020-Dec-14	A/Chicken/Netherlands/20019879-001005/2020	Wageningen Bioveterinary Research	Wageningen Bioveterinary Research	Beerens, Nancy; Harders, Frank; Pritz-Verschuren, Sylvia; Roose, Marit; Venema, Sandra; Germeraad, Evelien; Engelsma, Marc; Heutink, Rene
EPI_ISL_9856775	Netherlands	2021-Oct-25	A/chicken/Netherlands/21037287-006010/2021	Wageningen Bioveterinary Research	Wageningen Bioveterinary Research	Beerens, Nancy; Harders, Frank; Pritz-Verschuren, Sylvia; Roose, Marit; Venema, Sandra; Germeraad, Evelien; Engelsma, Marc; Heutink, Rene; Luca, Bordes

EPI_ISL_711055	Netherlands	2020-Dec-14	A/chicken/Netherlands/20019879-001005/2020	Wageningen Bioveterinary Research	Wageningen Bioveterinary Research	Beerens, Nancy; Harders, Frank; Pritz-Verschuren, Sylvia; Roose, Marit; Germeraad, Evelien; Engelsma, Marc; Bossers, Alex; Heutink, Rene
EPI_ISL_18458069	Poland	2021-Nov-17	A/duck/Poland/H1993_21RS3290-14/2021	National Veterinary Research Institute	Istituto Zooprofilattico Sperimentale Delle Venezie	Smietanka, K.; Swieton, E.;Zecchin, B.;Pastori, A.;Fusaro, A.;Schivo, A.;Salviato, A.;Palumbo, E.;Giussani, E.;Monne, I.;Terregino, C.
EPI_ISL_13370920	United Kingdo	2022-Apr-04	A/domestic_duck/England/040831/2022	Animal and Plant Health Agency (APHA)	Animal and Plant Health Agency (APHA)	
EPI_ISL_17884628	Czech Repub	2023-May-05	A/common_tern/Czech_Republic/8807-5/2023	State Veterinary Institute Prague	State Veterinary Institute Prague	Alexander,Nagy;Lenka,Cernikova;Martina,Stara
EPI_ISL_683592	Italy	2020-Nov-21	A/Eurasian_wigeon/Italy/20VIR7301-206/2020	Istituto Zooprofilattico Sperimentale delle Venezie, EU/OIE/Reference Laboratory and FAO Reference Centre for AI and ND	Istituto Zooprofilattico Sperimentale Delle Venezie	Zecchin, B.; Fusaro, A.; Pastori, A.; Milani, A.; Salviato, A.; Schivo, A.; Monne, I.; Terregino, C.
EPI_ISL_632316	Netherlands	2020-Nov-01	A/eurasian curlew/Netherlands/20016890-001/2020	Wageningen Bioveterinary Research	Wageningen Bioveterinary Research	Beerens, Nancy; Harders, Frank; Verschuren-Pritz, Sylvia; Roose, Marit; Germeraad, Evelien; Engelsma, Marc; Bossers, Alex; Heutink, Rene
EPI_ISL_11798579	Denmark	2021-Nov-01	A/greylag_goose/Denmark/24343-1.02/2021-11-01	Statens Serumm Institute	Statens Serum Institute	Charlotte Hjulsager, Yuan Liang
EPI_ISL_711059	Netherlands	2020-Dec-08	A/greylag goose/Netherlands/20019685-002/2020	Wageningen Bioveterinary Research	Wageningen Bioveterinary Research	Beerens, Nancy; Harders, Frank; Pritz-Verschuren, Sylvia; Roose, Marit; Germeraad, Evelien; Engelsma, Marc; Bossers, Alex; Heutink, Rene
EPI_ISL_632314	Netherlands	2020-Oct-28	A/greylag goose/Netherlands/20016582-004/2020	Wageningen Bioveterinary Research	Wageningen Bioveterinary Research	Beerens, Nancy; Harders, Frank; Verschuren-Pritz, Sylvia; Roose, Marit; Germeraad, Evelien; Engelsma, Marc; Bossers, Alex; Heutink, Rene
EPI_ISL_7054529	Sweden	2021-Nov-08	A/greylag goose /Sweden/SVA211111SZ0376/FB004497/M-2021	Swedish Veterinary Agency (SVA)	Swedish Veterinary Agency (SVA)	
EPI_ISL_8515482	Czech Repub	2021-Nov-28	A/grey heron/Czech_Republic/23608-1K/2021	State Veterinary Institute Prague	State Veterinary Institute Prague	
EPI_ISL_16548638	Belgium	2022-Oct-01	A/Phasianus_colchicus/Belgium/11704-0003/2022	Sciensano - Animal Infectious Diseases	Sciensano, Department of Animal Infectious Diseases	Van Borm, Steven; Roupie, Virginie; Hostyn, Pierre; Lambrecht, Benedicte; Steensels, Mieke
EPI_ISL_17716825	Czech Repub	2023-May-11	A/black-headed_gull/Czech_Republic/7334-5/2023	State Veterinary Institute Prague	State Veterinary Institute Prague	Alexander,Nagy;Lenka,Cernikova;Martina,Stara
EPI_ISL_13370416	United Kingdo	2021-Nov-30	A/Black-headed_gull/England/064433/2021	Animal and Plant Health Agency (APHA)	Animal and Plant Health Agency (APHA)	
EPI_ISL_18094395	Russian Fede	2023-Jul-28	A/black-headed gull/Leningrad_region/RII-WD263M/2023	WHO National Influenza Centre Russian Federation	WHO National Influenza Centre Russian Federation	
EPI_ISL_6596216	Sweden	2021-Nov-02	A/barnacle goose/Sweden/SVA211102SZ0402/FB004395/M-2021	Swedish Veterinary Agency (SVA)	Swedish Veterinary Agency (SVA)	
EPI_ISL_13370613	United Kingdo	2021-Dec-17	A/chicken/England/073008/2021	Animal and Plant Health Agency (APHA)	Animal and Plant Health Agency (APHA)	
EPI_ISL_10261376	Germany	2022-Jan-24	A/pigeon/Germany-NW/AI00951/2022	Chemisches- und Veterinäruntersuchungsamt Westfalen	Friedrich-Loeffler-Institut	
EPI_ISL_6931008	Poland	2021-Nov-03	A/domestic_goose/Poland/H1931-T1/2021	National Veterinary Research Institut Poland, PIWet-PIB	National Veterinary Research Institut Poland, PIWet-PIB	E. Swieton, K. Smietanka
EPI_ISL_18123942	Finland	2023-Jul-19	A/black-headed-gull/Finland/8145/2023	Finnish Food Authority	Finnish Institute for Health and Welfare, THL	Kantala, Tuija; Fusaro, Alice; Gadd, Tuija; Gallo, Monica; Ikonen, Niina; Kareinen, Lauri; Kauppinen, Ari; Tammiranta, Niina; Lindh, Erika
EPI_ISL_5323346	Czech Repub	2021-Sep-27	A/goose/Czech Republic/18520-1/2021	State Veterinary Institute Prague	State Veterinary Institute Prague	Alexander,Nagy; Lenka,Cernikova; Martina,Stara
EPI_ISL_9160206	Belgium	2021-Nov-21	A/Anser_albifrons/Belgium/15465_0010/2021	Sciensano - Animal Infectious Diseases	Sciensano, Department of Animal Infectious Diseases	Van Borm, Steven; Roupie, Virginie; Lambrecht, Benedicte; Mathijs, Elisabeth; Steensels, Mieke
EPI_ISL_632315	Netherlands	2020-Nov-02	A/eurasian teal/Netherlands/20016896-013/2020	Wageningen Bioveterinary Research	Wageningen Bioveterinary Research	Beerens, Nancy; Harders, Frank; Verschuren-Pritz, Sylvia; Roose, Marit; Germeraad, Evelien; Engelsma, Marc; Bossers, Alex; Heutink, Rene
EPI_ISL_7996371	Netherlands	2021-Dec-20	A/turkey/Netherlands/21040980-001005/2021	Wageningen Bioveterinary Research	Wageningen Bioveterinary Research	Beerens, Nancy; Harders, Frank; Pritz-Verschuren, Sylvia; Roose, Marit; Venema, Sandra; Germeraad, Evelien; Engelsma, Marc; Heutink, Rene
